# Reflection on modern methods: years of life lost due to premature mortality—a versatile and comprehensive measure for monitoring non-communicable disease mortality

**DOI:** 10.1093/ije/dyy254

**Published:** 2019-01-09

**Authors:** Ramon Martinez, Patricia Soliz, Roberta Caixeta, Pedro Ordunez

**Affiliations:** 1 Department of Non-Communicable Diseases and Mental Health; 2 Department of Evidence and Intelligence for Action in Health, Pan American Health Organization, Washington, DC, USA

**Keywords:** Mortality, premature, epidemiological method, non-communicable diseases, public health surveillance

## Abstract

The analysis of causes impacting on premature mortality is an essential function of public health surveillance. Diverse methods have been used for accurately assessing and reporting the level and trends of premature mortality; however, many have important limitations, particularly in capturing actual early deaths. We argue that the framework of years of life lost (YLL), as conceptualized in disability-adjusted life-years (DALYs), is a robust and comprehensive measure of premature mortality. Global Burden of Disease study is systematically providing estimates of YLL; however, it is not widely adopted at country level, among other reasons because its conceptual and methodological bases seem to be not sufficiently known and understood. In this paper, we provide the concepts and the methodology of the YLL framework, including the selection of the loss of function that defines the time lost due to premature deaths, and detailed methods for calculating YLL metrics. We also illustrate how to use YLL to quantify the level and trends of premature non-communicable disease (NCD) mortality in the Americas. The tutorial style of the illustrative example is intended to educate the public health community and stimulate the use of YLL in disease prevention and control programmes at different levels.

**Key Messages**

- YLL due to premature deaths is an accurate measure for assessing the impact of diseases, injuries and risk factors on premature mortality.
- YLL is easy to calculate and understand, and the YLL metrics enable comprehensive premature mortality analysis.
- YLL is a valuable measure for public health surveillance, particularly for quantifying the level and trends of premature mortality, identification of leading causes of premature deaths and monitoring the progress of YLL as a key indicator of population health.

## Introduction

Efforts to accurately measure premature mortality are not new in epidemiology. However, the rise of non-communicable diseases (NCD) to epidemic levels and the need for monitoring its impact on premature mortality^1^^,^^2^ have currently emphasized its relevance for public health surveillance. Indeed, different methods have been used for reporting premature mortality: (i) proportion of premature deaths under a selected age threshold; (ii) age-standardized death rates under a defined age range; (iii) years of potential life lost (YPLL) between ages at death and a selected age cut-off^3^; (iii) probability of survival at a specified age, for example 70,^4^ estimated from life tables; and (iv) probability of dying between an exact age range, derived from the life table method.^5^ The normative age threshold, embedded in these measures, leads them to fail in capturing avoidable deaths at ages outside the selected age range, which is considered an important limitation.

Years of life lost (YLL) due to premature mortality, as developed by the Global Burden of Disease, Injuries and Risk Factor (GBD) study,^6^ overcomes the issue related to the arbitrary selection of age threshold by basing its metrics on time lost rather than number of deaths, and calculating time lost based on the potential maximum life span of an individual at each age. YLL is being systematically estimated by the GBD study to support public health planning and guide public health policy and programmes at global, national and subnational levels. However, it is not widely adopted, among other reasons because its conceptual and methodological bases seem to be not sufficiently known and understood.

The aim of this paper is to provide the essential concepts and the methodology of the YLL, including the selection of loss functions that define the time lost due to premature deaths, and detailed methods for calculating YLL metrics. We illustrate how to use YLL to quantify the level and trends of premature NCD mortality in the Americas, with the ultimate purpose of contributing to the education of the public health community, and stimulate its use in disease prevention and control programmes at different levels.

## YLL conceptual bases and methodology

YLL due to premature deaths were conceptualized by the GBD study^6^ as the time-based measure to estimate the loss of years of life associated with a death. It is the mortality component of the disability-adjusted life-years (DALY),^7^ and it relies on the concept of time lost as the most appropriate measurement of the impact of diseases, injuries and risk factors on premature mortality. In the GBD,^7^ a premature death is defined as a death that occurred before the potential maximum life expectancy observed at the age of the person who died, and the standard expected years of life lost method was chosen to measure the duration of time lost due to premature mortality. Thus, the calculation of time lost is based on the difference between age at death and the standard life expectancy (SLE) at that age; where SLE is a time loss function on the age at death which quantifies the years of life lost due to early death.

As a measure of the burden of premature mortality, YLL has the following advantages: (i) it avoids arbitrary judgements about age cut-offs, which are never methodologically justifiable, and exclusions of older population groups; (ii) all deaths imply the loss of some potential years of life, which means that deaths at all ages contribute to the quantification of the burden of premature mortality; (iii) YLL places greater weight on deaths that occur at younger ages; and (iv) a death at a given age represents the same amount of years of life lost irrespective of the location where it occurred, keeping the egalitarian nature of YLL.^7^ The main data source for YLL is the vital statistics and mortality information system, so the quality of YLL will depend ultimately on the quality of mortality statistics, assessed by the level of registration coverage, timeliness, completeness and accuracy of underlying causes of death diagnosis and coding. The GBD study addresses the issue of mortality data quality, conducting a set of standard methods for correcting registered death data by missing values and under-registration, and redistributing ill-defined and ‘garbage codes’ causes to well-defined and public health relevant causes which are mapped to the GBD cause list. Assessment of vital registration systems and application of standard methods to overcome data quality issues are always required before any mortality analysis.

## The SLE as the loss function on the age at death

The SLE, a fundamental element to quantify YLL, is intended to represent the potential maximum life span of an individual at a given age, who is not exposed to avoidable health risks or severe injuries and receives appropriate health services.^7^ Its selection has evolved across editions of the GBD studies and the World Health Organization (WHO) Global Health Estimates (GHE) as shown in SupplementaryTable 1, available as Supplementary data at *IJE* online. In summary, in GBD 1990, the SLE was based on the highest observed life expectancy at the time based on Coale–Demeny Model Life Table West.^8^ Different SLEs were used for males and females,^7^^,^^9^ and age-weighted and age-discounted as social preferences were considered. In the GBD 2010 study, a new normative standard life table for males and females was developed, based on the lowest observed death rate for any age group in countries of more than 5 million population.^6^ In the GBD 2013 study, the standard life table was updated^10^ and its SLE has been used in subsequent GBD studies.

In the WHO GHE, the SLE was based on the highest national life expectancy projected for the year 2050,^11^ considering that it is not appropriate to set the normative loss of years of life in terms of currently observed death rates, since even for the lowest observed death rates there are a proportion of deaths which are preventable or avertable. Whereas this may still not represent the ultimate achievable human life spans, it represents a set of life spans which are thought likely to be achieved by a substantial number of people who are alive today. As shown in Supplementary Table 2, available as Supplementary data at *IJE* online, at any age, even above 100 years, some YLL accrue.

## Calculation of YLL due to premature mortality

YLL at the individual level is calculated by subtracting the age at death from the SLE at that age. For instance, if the SLE at age 72 is a further 14 years, then someone dying at that age from a specified cause will have lost 14 years of life. At the population level, the number of YLL is calculated as the number of deaths due to a cause at a certain age, sex, place and time, multiplied by the SLE at age of death.

## The absolute number of YLL

The number of YLL due to a cause *c*, in a population of sex *s*, age *a* and period *t*, is the basic metric of YLL. It can be calculated by the formula:

YLL(c, s, a, t)=D(c, s, a, t)×SLE(a)

where *D(c, s, a, t)* is the number of deaths due to the cause *c*, sex *s* and age *a*, and period *t SLE(a)* is the standard life expectancy at age *a.* This metric quantifies the absolute number of YLL due to premature deaths in certain population.

## Calculation of YLL as proportion

YLL for a given cause can also be measured as proportion of all-causes YLL by dividing the number of cause-specific YLL by the number of all-causes YLL, usually expressed in percentages, as expressed by the formula:

YLL proportion(c, s, a, t)=YLL(c, s, a, t)/YLL(s, a, t)×100%

where *YLL(c, s, a, t)* is the number of YLL due to cause *c*, sex *s* and age *a*, and period *t YLL(s, a, t)* is the number of all-causes YLL at sex *s*, age *a* and period *t.*

The proportion of cause-specific YLL provides insight about the relative burden of causes of premature deaths. It is easy to calculate and interpret, requiring only the number of YLL per cause of death. It is appropriate for ranking causes of deaths in a specific population group and period of time. It should not be used for comparison across population groups or over time, because it does not account for population size and changes in age structure.

## Calculation of YLL rates

YLL rate is a more meaningful metric for cross-population comparisons as it accounts for the population size. YLL rate due to cause *c*, in the population of sex *s* and age *a*, and time *t* can be calculated by the formula:

YLL rate(c, s, a, t)=YLL(c, s, a, t)/P(s, a, t)×100 000 population

where *YLL(c, s, a, t)* is the number of YLL due to cause *c*, in population of sex *s* and age *a*, and period *t*, *P(s, a, t*) is the population size at sex *s*, age *a* and period *t.*

YLL rate provides a relative quantification of the magnitude of the impact of diseases, injuries and risk factors on the premature mortality in the population, but does not control for differences in the population age distribution. It is useful for comparison among sex and age groups, for example age-sex-specific YLL rates. However, all-ages YLL rates are not appropriate for comparison across population groups and over time, because they are not adjusted by the population age structure.

## Calculation of age-standardized YLL rates

Limitations of all-ages YLL rates are solved by applying the direct age-standardization method^12^ using a standard population. The age-standardized YLL rate (ASYR) due to cause *c*, in the population of sex *s* and period *t*, can be calculated by the formula:

$$ASYR(c,s,t)=\;\underset{a}{\sum }YLLrate(c,s,a,t)\times W(a)$$

where *YLL rate (c, s, a, t)* is the YLL rate due to cause *c*, in population of sex *s* and age *a*, and period *t W(a)* is the standard population weight at age *a.*

ASYR controls for population age distribution, which is important for ensuring comparability across groups with different population age structures. This metric is appropriate for assessing the level and trends of premature mortality across several population groups and over time. ASYR is interpreted as the number of YLL due to premature mortality per population as if the specified group has the same population age distribution of the standard population. Furthermore, all YLL metrics (number, proportion and rate) can be calculated by analytical dimensions such as socioeconomic status, ethnicity. Rates can also be adjusted for other confounders if needed. The SLE and the standard population used in the calculation of the YLL should be explicitly reported, to ensure correct interpretation of YLL estimates and further comparability across studies.

## Illustrative example: quantifying the level and trends of YLL due to premature NCD mortality in the Americas

This example is meant to illustrate the method for calculating YLL, and how to analyse premature mortality in the context of the public health practice. We used the comprehensive GHE 2015 dataset that includes death estimates and their 95% uncertainty intervals (95% UI) by cause-of-death, age, sex and year for WHO Member States with population size of 90 000 and over.^13^ GHE 2015 data sources and estimation methods are published elsewhere.^14^ In summary, data from national vital statistics and mortality information systems reported to WHO by national authorities are the main source. Mortality information is corrected by missing sex and age, and deaths are rescaled by sub-registration. Cause-of-death data quality issues due to diagnostic and coding accuracy are also corrected using standard procedures. Deaths with underlying causes of deaths coded to ill-defined and garbage codes are redistributed to well-defined causes and mapped to the GBD list of causes. These procedures are intended to overcome data quality issues and improve accuracy of mortality measures and the utility of mortality analysis results for programme and policy development; 33 countries of the Americas (Supplementary Table 3, available as Supplementary data at *IJE* online), representing more than 98% of the total population, were included in the analysis. Four major NCD deaths were defined as those from cardiovascular diseases (CVD, I00-I99), cancer (C00-C97), diabetes (E10-E14) and chronic respiratory diseases (J30-J98), according to the GHE 2015 cause-of-death list.^14^

## Step 1: computation of YLL metrics

The first step of the analysis is the computation of YLL metrics (number of deaths, YLL, age-and-sex-specific YLL rates, and ASYR). Therefore, the annual number of deaths and 95% UI from the four major NCDs by age and sex for the Region of the Americas from 2000 to 2015 were calculated by summing deaths from CVD, cancer, diabetes and chronic respiratory diseases. YLL were calculated using the SLE from the WHO GHE. Age-and-sex-specific YLL rates were calculated using the mid-year population estimates from the World Population Prospects, 2017 Revision,^15^ and ASYR was computed by direct method^12^ using the WHO world standard population.^16^ For each YLL metric, the 95% UIs were propagated from the 95% UIs of the estimated deaths. Table 1 illustrates the calculation of YLL metrics for 2015. The SLE, population size and standard population weights by age are also included, to facilitate understanding of the calculations.

**Table 1.** Premature mortality from four major non-communicable diseases, in both sexes, combined populations in the Region of the Americas, 2015

**Age group**	**Deaths (a)**	**Deaths (95% LL) (b)**	**Deaths (95% UL) (c)**	**SLE (d)**	**YLL (e) = (a) × (d)**	**YLL (95% LL) (f) = (b) × (d)**	**YLL (95% UL) (g) = (c) × (d)**	**Population (h)**	**YLL rate (i) = (e)/ (h) ×100 000**	**YLL rate (95% LL) (j) = (f)/(h)× 100 000**	**YLL rate (95% UL) (k) = (g)/(h) ×100 000**	**Std pop weight (l)**	**ASYR (m) = (i)×(l)**	**ASYR (95% LL) (n) = (j)×(l)**	**ASYR (95% UL) (o) = (k)×(l)**
0–4	10 109	7006	13 351	89.41	903 870	626 432	1 193 730	74 910 767	1206.6	836.2	1593.5	0.0886	106.9	74.1	141.2
5–9	4844	3812	6160	84.52	409 448	322 219	520 654	75 955 736	539.1	424.2	685.5	0.0869	46.8	36.9	59.6
10–14	4925	4012	6073	79.53	391 663	319 040	482 998	77 055 947	508.3	414.0	626.8	0.0860	43.7	35.6	53.9
15–19	8965	7388	10 972	74.54	668 288	550 709	817 827	78 750 920	848.6	699.3	1038.5	0.0847	71.9	59.2	88.0
20–24	12 334	10 275	14 987	69.57	858 048	714 823	1 042 649	79 725 807	1076.2	896.6	1307.8	0.0822	88.5	73.7	107.5
25–29	17 215	14 484	20 710	64.60	1 112 097	935 689	1 337 884	77 478 740	1435.4	1207.7	1726.8	0.0793	113.8	95.8	136.9
30–34	26 836	23 009	31 628	59.63	1 600 214	1 372 043	1 885 955	73 714 765	2170.8	1861.3	2558.4	0.0761	165.2	141.6	194.7
35–39	39 472	34 312	45 869	54.67	2 157 925	1 875 858	2 507 676	68 415 366	3154.2	2741.9	3665.4	0.0715	225.5	196.0	262.1
40–44	64 154	56 615	73 399	49.73	3 190 381	2 815 472	3 650 140	63 794 631	5001.0	4413.3	5721.7	0.0659	329.6	290.8	377.1
45–49	105 182	93 614	119 276	44.81	4 713 227	4 194 851	5 344 778	60 369 057	7807.4	6948.7	8853.5	0.0604	471.6	419.7	534.8
50–54	181 543	163 157	203 752	39.92	7 247 186	6 513 220	8 133 772	57 785 174	12 541.6	11 271.4	14 075.9	0.0537	673.5	605.3	755.9
55–59	266 676	241 132	297 057	35.07	9 352 322	8 456 516	10 417 776	51 389 071	18 199.0	16 455.9	20 272.4	0.0455	828.1	748.7	922.4
60–64	342 106	310 038	379 983	30.25	10 348 705	9 378 656	11 494 482	43 427 629	23 829.8	21 596.1	26 468.1	0.0372	886.5	803.4	984.6
65–69	414 043	376 015	458 633	25.49	10 553 962	9 584 628	11 690 557	34 099 240	30 950.7	28 108.0	34 283.9	0.0296	916.1	832.0	1014.8
70–74	464 968	422 132	515 202	20.77	9 657 395	8 767 677	10 700 745	24 645 889	39 184.6	35 574.6	43 418.0	0.0221	866.0	786.2	959.5
75–79	497 750	451 521	551 734	16.43	8 178 032	7 418 496	9 064 997	17 733 596	46 116.0	41 833.0	51 117.6	0.0152	701.0	635.9	777.0
80–84	527 106	479 244	582 163	12.51	6 594 092	5 995 339	7 282 855	12 003 732	54 933.7	49 945.6	60 671.6	0.0091	499.9	454.5	552.1
85+	944 280	859 498	1 041 327	7.60	7 176 528	6 532 184	7 914 089	11 519 802	62 297.3	56 704.0	68 699.9	0.0063	392.5	357.2	432.8
Total	3 932 508	3 557 264	4 372 276		85 113 383	76 373 852	95 483 564	982 775 869	8660.5	7771.2	9715.7		7426.9	6646.7	8354.8

## Step 2: what is the burden of premature NCD mortality?

An overview using the annual number of YLL due to premature NCD deaths is a good starting point. In 2015, the four major NCDs accounted for a total of 3 932 508 deaths (95% UI 3 557 264–4 372 276), representing 85 113 383 YLL (76 373 852–95 483 564) due to premature deaths. In men, YLL was 46 272 426 (41 649 979–51 739 771), an excess of 9 431 465 YLL compared with women with 38 840 961 YLL (34 723 873–43 743 793). ASYR was 7426.9 (95% UI 6646.7–8354.8) per 100 000 population, higher in men with 8756.0 (7870.5–9803) YLL per 100 000 than in women with 6272.0 (5583.4–7095.5) YLL per 100 000. This means that region-wide, men died at earlier ages than women, thus losing more years of life.

## Step 3: how premature NCD mortality is distributed by age group and sex

**Figure 1.**
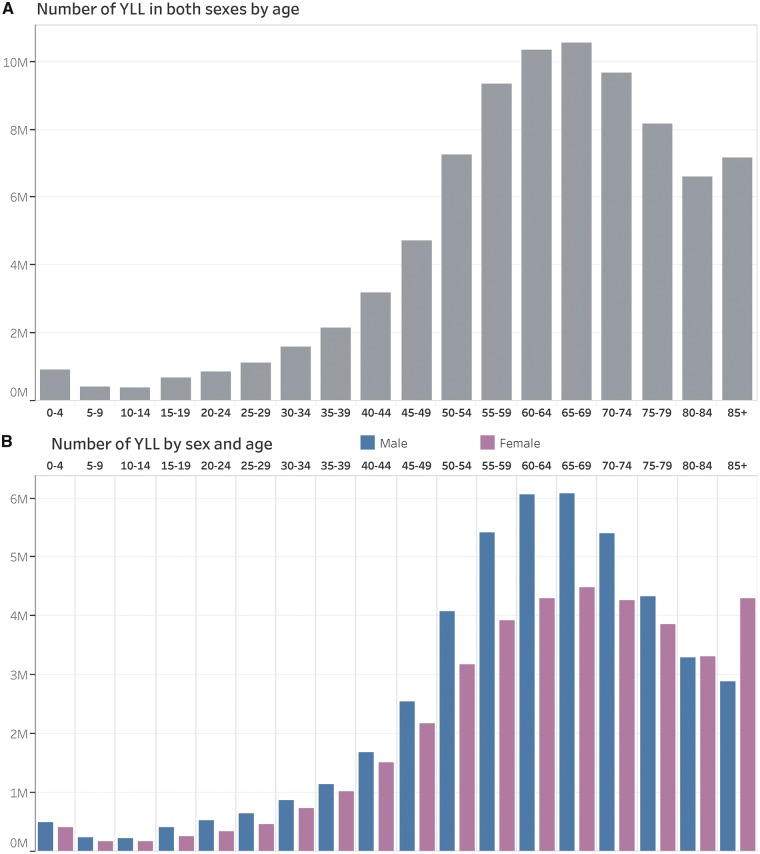
Absolute number of YLL by age in both sexes combined, males and females, in the Region of the Americas, 2015.

The YLL distribution pattern by age and sex can be explored and analysed using absolute number of YLL and age-and-sex-specific YLL rates. Figure 1 A shows that age groups 50–54, 55–59, 60–64, 65–69, 70–74 and 75–79 had the highest number of YLL, which is consistently higher in men compared with women across age groups, except at ages of 80–84 and 85 and over (Figure 1B).

**Figure 2.**
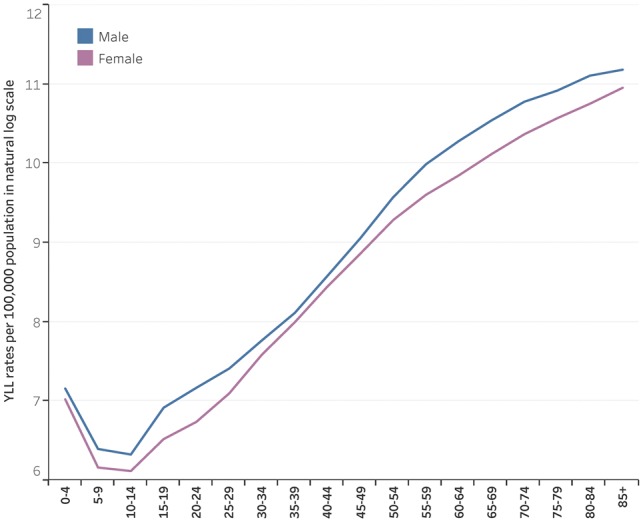
Age-specific YLL rates per 100 000 population (in natural logarithm scale) by sex, Region of the Americas, 2015.

The age-and-sex-specific YLL rates are high at the early ages of 0–4 years, they drop at ages 5–9 and 10–14, increase from 15–19 to 64–69, then slightly slow down at ages 70–74 and 85+ years. Males have a higher burden of premature mortality than women, with pronounced differences in youth and adolescents and at adult ages of 50–54 years and over (Figure 2 ).

## Step 4: what are the leading causes impacting on the premature NCD mortality?

The identification of leading causes impacting on premature deaths can provide insights about the underlying diseases processes at early deaths, the performance of health systems and the effectiveness of preventive interventions. Cause-specific YLL proportions, crude and age-and-sex-specific YLL rates, and ASYR are useful metrics for this purpose. In this example, ASYR is the most appropriate metric because we are comparing leading causes in all ages and both sexes combined in two periods, thus avoiding bias from the effects of changes in the population size and age structure of the population. We rank NCD causes at the regional level in 2000 and 2015, including percent change in ASYR from 2000 to 2015 (Table 2 ).

**Table 2.** Leading 25 non-communicable disease causes for age-standardized YLL rates (ASYR) per 100 000 population for both sexes combined in 2000 and 2015, and percent change in ASYR from 2000 to 2015, Region of the Americas

2000			2015			
Rank	Leading NCD causes	ASYR	Rank	Leading NCD causes	ASYR	Percent change 2000–15
1	Ischaemic heart disease	2548.20	1	Ischaemic heart disease	1720.20	−32.5%
2	Stroke	1126.20	2	Stroke	723.7	−35.7%
3	Trachea, bronchus, lung cancers	711.9	3	Diabetes mellitus	668.4	−2.7%
4	Diabetes mellitus	686.8	4	Trachea, bronchus, lung cancers	513.9	−27.8%
5	Chronic obstructive pulmonary disease	626.2	5	Chronic obstructive pulmonary disease	503.3	−19.6%
6	Cirrhosis of the liver	523.1	6	Cirrhosis of the liver	448.3	−14.3%
7	Congenital heart anomalies	345	7	Kidney diseases	325.5	−4.2%
8	Kidney diseases	339.7	8	Alzheimer disease and other dementias	270.5	66.8%
9	Breast cancer	304.4	9	Congenital heart anomalies	255.2	−26.0%
10	Colon and rectum cancers	296	10	Colon and rectum cancers	253.1	−14.5%
11	Cardiomyopathy, myocarditis, endocarditis	258.8	11	Breast cancer	253	−16.9%
12	Stomach cancer	209.9	12	Drug use disorders	202.7	67.7%
13	Lymphomas, multiple myeloma	206.6	13	Cardiomyopathy, myocarditis, endocarditis	173.3	−33.0%
14	Leukaemia	193.9	14	Hypertensive heart disease	168.6	1.8%
15	Hypertensive heart disease	165.6	15	Leukaemia	166.5	−14.1%
16	Alzheimer disease and other dementias	162.2	16	Stomach cancer	161.4	−23.1%
17	Pancreas cancer	150	17	Lymphomas, multiple myeloma	157	−24.0%
18	Prostate cancer	147.5	18	Pancreas cancer	145.1	−3.3%
19	Alcohol use disorders	146.5	19	Liver cancer	132.6	7.7%
20	Cervical cancer	141.2	20	Brain and nervous system cancers	132.3	−4.5%
21	Brain and nervous system cancers	138.6	21	Prostate cancer	122	−17.3%
22	Liver cancer	123.1	22	Cervical cancer	112	−20.7%
23	Drug use disorders	120.9	23	Alcohol use disorders	110.5	−24.6%
24	Oesophagus cancer	99.4	24	Oesophagus cancer	82.3	−17.2%
25	Ovary cancer	80.1	25	Ovary cancer	68.2	−14.9%

Causes were rated based on age-standardized YLL rates (ASYR). List of causes included level 3 causes and those causes from level 2 with no cause category al level 3.

In 2015, ischaemic heart disease; stroke; diabetes mellitus; trachea, bronchus, lung cancers; and chronic obstructive pulmonary disease were the top five diseases impacting on years of life lost due to premature NCD mortality region-wide. Important reductions of ASYR per 100 000 population have been observed in most of the leading 25 NCD causes from 2000 to 2015. This means that deaths from those causes have been avoided and postponed to older ages, probably due to improved health care access and quality and successful preventive interventions. However, ASYR increased 67.7% in drug use disorders (moving from position 23 in 2000 to 12 in 2015), 66.8% in Alzheimer disease and other dementias (moving from position 16 in 2000 to 8 in 2015), and 7.4% in liver cancer. The epidemiological shift of these three causes requires a thorough investigation and focused public health attention.

## Step 5: what are the countries with the higher levels of premature NCD mortality?

**Figure 3.**
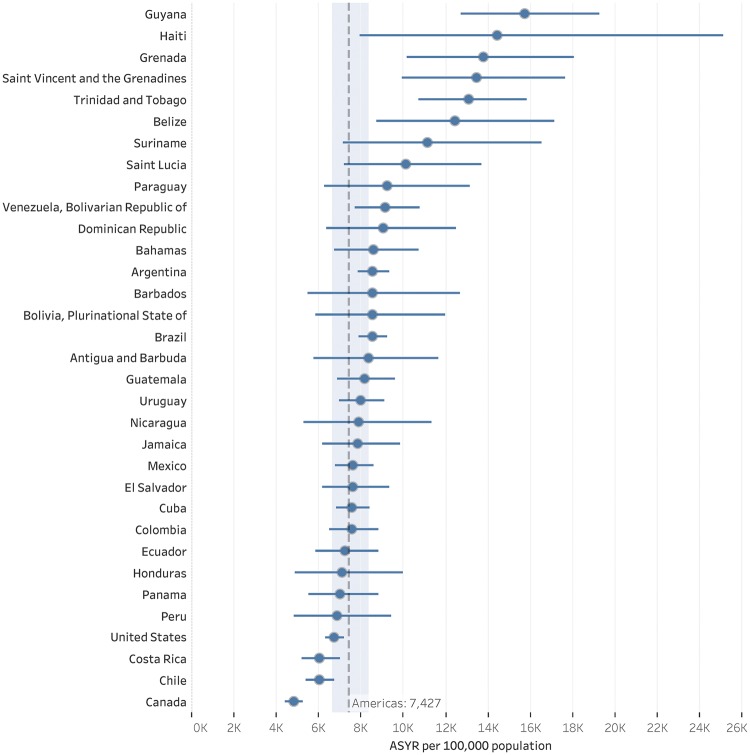
Age-standardized YLL rates (ASYR) per 100 000 population and 95% UIs in both sexes combined. in countries of the Americas, 2015. Dot (•) represents ASYR estimates, and line (—) represents the 95% uncertainty intervals. Dashed line represents the ASYR per 100 000 population in the Region of the Americas, and the light blue band represents its 95% UI.

The analysis of the level of YLL across countries provides useful information to explore disparities and its association with socioeconomic indicators, and eventually to guide public health actions. The ASYR is the appropriate metric for assessing the level of premature mortality across countries. Figure 3 shows a tremendous disparity among countries of the Americas, where Guyana, Haiti, Grenada, Saint Vincent and the Grenadines, and Trinidad and Tobago have the highest burden of premature NCDs mortality. These countries have age-standardized YLL rates per 100 000 population almost four times higher than Canada, the country with lowest ASYR in the region.

## Step 6: what are the trends of premature NCD mortality?

A trends analysis of ASYR from the four major NCDs by sex at regional level from 2000 to 2015 was conducted using Joinpoint regression methods.^17^ It provided estimates of the annual percent change (APC) and the average annual percent change (AAPC) with their 95% UIs, as trend measures.

**Figure 4.**
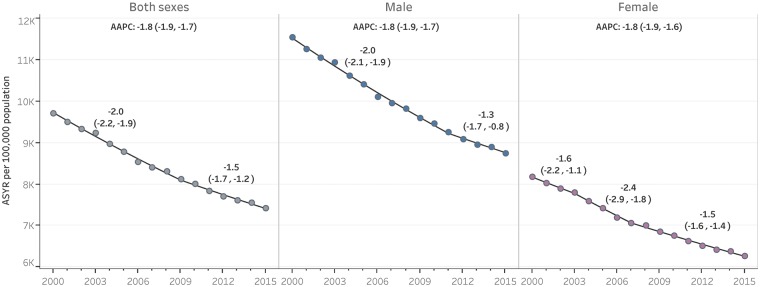
Trends of ASYR per 100 000 population from the four major NCDs in both sexes, males and females, annual percent change (APC) per segment and average annual percent change (AAPC), Region of the Americas, 2000–15. APC refers to annual percent change in each trend segment. AAPC refers to average annual percent change in the whole time series from 2000 to 2015; 95% uncertainty intervals (95% UIs) for APC and AAPC are provided between parentheses. Dot (•) represents age-standardized YLL rates (ASYR), and line (—) represent the regression model values, both per 100 000 population. Estimates of APC and AAPC and their 95% UIs, and the regression model, were obtained from Joinpoint regression analysis.

The ASYR in both sexes declined at an AAPC of −1.8% (95% UI −1.9%, −1.7%), a 27% reduction from 2000 to 2015. ASYRs are higher in males than in females; however, they are declining at a similar rate (Figure 4 ). In both sexes combined, the reduction rate slowed down to −1.5% (−1.7%, −1.2%) from 2009 to 2015. In males, APC decreased −1.3% (−1.7%, -0.8%) from 2011 to 2015 and in women, it decreased −1.5% (−1.6%, −1.4%) from 2011 to 2015 (Supplementary Table 5, available as Supplementary data at *IJE* online).

Trends analysis provides useful information for assessing the progress of ASYR due to premature NCD mortality toward a defined target. For instance, the Sustainable Development Goal’s Target 3.4 states ‘by 2030, reduce by one-third premature mortality from non-communicable diseases through prevention and treatment and promote mental health and well-being’.^18^ To reach this target, at least an AAPC of −1.6% is required. If the Region of the Americas maintain the average rate of decline [AAPC of −1.5% (−1.7%, −1.2%)] observed from 2010 to 2015, it will be unlikely to reach the target, so more sound efforts are needed.

## Final remarks

The YLL is a versatile, accurate and comprehensive measure of premature mortality. It is able to reflect the mortality patterns dominated by underlying disease processes occurring at early deaths, most of which could be postponed to older ages or are avoidable with effective public health interventions. Given the simplicity of calculation and ease of comprehension, the YLL becomes a key indicator for assessing and guiding the progress of public health policies and interventions. The illustrated example demonstrates the usability of YLL for measuring and monitoring the level and trends of NCDs impacting on premature mortality.

We present the conceptual bases and methodology of years of life lost due to premature mortality, with emphasis on: (i) the standard life expectancy as a loss function on age to determine years lost from early deaths; and (ii) the methods to calculate four main metrics of years of life lost due to premature mortality. With an illustrative example, we demonstrate that it is feasible to apply YLL methodology in the public health practice, particularly in the surveillance of non-communicable diseases.

**Conflict of interest:** None declared. The content, findings and conclusions in this report are solely responsibility of the authors and do not necessarily represent the official position of the Pan American Health Organization.

## Supplementary Material

dyy254_Supplementary_Data
