# Supplementary material for: Reflection on modern methods: years of life lost due to premature mortality—a versatile and comprehensive measure for monitoring non-communicable disease mortality
Source: Int J Epidemiol. 2019 Jan 9;48(4):1367–76. doi: 10.1093/ije/dyy254 (PMC6693813; doi:10.1093/ije/dyy254)
Supplement: dyy254_Supplementary_Data [file dyy254_supplementary_data.docx]

## Supplementary Appendix

This document provides supplementary material for the manuscript **Years of life lost due to premature mortality, conceptual bases and methodology, and application for monitoring noncommunicable disease mortality in the Americas**. The content of this document is intended to provide additional data and information to complement the content of the manuscript.

## Years of life lost due to premature mortality, conceptual bases and methodology, and application for monitoring noncommunicable disease mortality in the Americas

Ramon Martinez-Piedra* (1), Patricia Soliz (1), Roberta Caixeta (1), and Pedro Ordunez (1)

1. Department of Non-Communicable Diseases and Mental Health, Pan American Health Organization. Washington, D.C. USA.

*Corresponding author: Department of Non-Communicable Diseases and Mental Health, Pan American Health Organization. 525 23^rd^ St. NW. Washington, D.C. 22037. USA. Email: [martiner@paho.org](mailto:martiner@paho.org)

**Disclaimer**

The content, findings and conclusions in this report are solely responsibility of the authors and do not necessarily represent the official position of the Pan American Health Organization or any of the authors’ affiliated institutions.

Table of Contents

[**Supplementary Table 1.** Standard life expectancy for computing YLL used in Global Burden of Disease studies (GBD) and WHO Global Health Estimates (WHO GHE) 3](#_Toc514433436)

[**Supplementary Table 2.** World Health Organization Standard Life Expectancy by single-age for calculating YLL. 4](#_Toc514433437)

[**Supplementary Table 3.** List of WHO Member States with population size of 90,000 and over from the Region of the Americas. 5](#_Toc514433438)

[**Supplementary Table 4.** Premature mortality from four majors noncommunicable diseases in the Region of the Americas, 2015. 6](#_Toc514433439)

[**Panel A.** Both sexes 6](#_Toc514433440)

[**Panel B.** Males 7](#_Toc514433441)

[**Panel C.** Females 8](#_Toc514433442)

[**Supplementary Table 5.** Annual Percent Change (APC) and Average Annual Percent Change (AAPC) f the age-standardized YLL rates (ASYR) by sex, Region of the Americas. 9](#_Toc514433443)

[**Panel A.** ASYR and 95% uncertainty intervals (95% UI) for 2000 and 2015, AAPC and % change in ASYR in the period 2000-2015, and trends statistics 9](#_Toc514433444)

[**Panel B.** ASYR with 95% UI at the beginning and the end of segment, APC and % change in ASYR, and trends statistics 9](#_Toc514433445)

## **Supplementary Table 1. Standard life expectancy for computing YLL used in Global Burden of Disease studies (GBD) and WHO Global Health Estimates (WHO GHE)**

|  |  | **GBD 1990 age-weighted, discounted** | | **GBD 1990 no age-weights or discounting** | | **GBD 2010** | **GBD 2013-2016** | **WHO GHE** |
| --- | --- | --- | --- | --- | --- | --- | --- | --- |
| **age code** | **Age range** | **Male†** | **Female‡** | **Male†** | **Female‡** | **Total** | **Total** | **Total** |
| 0 | Neonatal (0-27 days) | 33.27 | 33.38 | 79.94 | 82.43 | 86.01 | 86.60 | 91.93 |
| 1 | Postneonetal (28-365 days) | 34.22 | 34.34 | 78.85 | 81.36 | 85.68 | 85.78 | 91.55 |
| 5 | 1-4 | 35.17 | 35.29 | 77.77 | 80.28 | 83.63 | 81.81 | 89.41 |
| 10 | 5-9 | 37.22 | 37.36 | 72.89 | 75.47 | 78.76 | 76.84 | 84.52 |
| 15 | 10-14 | 37.31 | 37.47 | 67.91 | 70.51 | 73.79 | 71.87 | 79.53 |
| 20 | 15-19 | 36.02 | 36.22 | 62.93 | 65.55 | 68.83 | 66.92 | 74.54 |
| 25 | 20-24 | 33.84 | 34.08 | 57.95 | 60.63 | 63.88 | 61.98 | 69.57 |
| 30 | 25-29 | 31.11 | 31.39 | 52.99 | 55.72 | 58.94 | 57.03 | 64.60 |
| 35 | 30-34 | 28.08 | 28.40 | 48.04 | 50.83 | 54.00 | 52.11 | 59.63 |
| 40 | 35-39 | 24.91 | 25.30 | 43.10 | 45.96 | 49.09 | 47.21 | 54.67 |
| 45 | 40-44 | 21.74 | 22.19 | 38.20 | 41.13 | 44.23 | 42.36 | 49.73 |
| 50 | 45-49 | 18.63 | 19.16 | 33.38 | 36.36 | 39.43 | 37.59 | 44.81 |
| 55 | 50-54 | 15.65 | 16.26 | 28.66 | 31.68 | 34.72 | 32.90 | 39.92 |
| 60 | 55-59 | 12.82 | 13.52 | 24.07 | 27.10 | 30.10 | 28.30 | 35.07 |
| 65 | 60-64 | 10.19 | 10.96 | 19.65 | 22.64 | 25.55 | 23.80 | 30.25 |
| 70 | 65-69 | 7.80 | 8.60 | 15.54 | 18.32 | 21.12 | 19.42 | 25.49 |
| 75 | 70-74 | 5.71 | 6.45 | 11.87 | 14.24 | 16.78 | 15.27 | 20.77 |
| 80 | 75-79 | 4.00 | 4.59 | 8.81 | 10.59 | 12.85 | 11.46 | 16.43 |
| 85 | 80-84 | 2.68 | 3.09 | 6.34 | 7.56 | 9.34 | 8.16 | 12.51 |
| 90 | 85+ | 1.37 | 1.23 | 3.82 | 3.59 | 5.05 | 5.53 | 7.60 |

Standard life expectancies are expresses in number of years by age group. **†** Life expectancy from model life table West 25 [1] for females was used for men (with life expectancy at birth of 80 years). **‡**Life expectancy from model life table West 26 [2] (with life expectancy at birth of 82.4 years for women. Life expectancy from WHO GHE is based on projected frontier period life expectancy and life table for year 2050 using UN World Population Prospects 2012[3]. GBD refers to Global Burden of Disease Study and WHO GHE refers to World Health Organization Global Health Estimates.

## Supplementary Table 2. World Health Organization Standard Life Expectancy by single-age for calculating YLL.

| **Age** | **SLE** | **Age** | **SLE** | **Age** | **SLE** |
| --- | --- | --- | --- | --- | --- |
| 0 | 91.94 | 35 | 57.15 | 70 | 23.15 |
| 1 | 91 | 36 | 56.16 | 71 | 22.23 |
| 2 | 90.01 | 37 | 55.17 | 72 | 21.31 |
| 3 | 89.01 | 38 | 54.18 | 73 | 20.4 |
| 4 | 88.02 | 39 | 53.19 | 74 | 19.51 |
| 5 | 87.02 | 40 | 52.2 | 75 | 18.62 |
| 6 | 86.02 | 41 | 51.21 | 76 | 17.75 |
| 7 | 85.02 | 42 | 50.22 | 77 | 16.89 |
| 8 | 84.02 | 43 | 49.24 | 78 | 16.05 |
| 9 | 83.03 | 44 | 48.25 | 79 | 15.22 |
| 10 | 82.03 | 45 | 47.27 | 80 | 14.41 |
| 11 | 81.03 | 46 | 46.28 | 81 | 13.63 |
| 12 | 80.03 | 47 | 45.3 | 82 | 12.86 |
| 13 | 79.03 | 48 | 44.32 | 83 | 12.11 |
| 14 | 78.04 | 49 | 43.34 | 84 | 11.39 |
| 15 | 77.04 | 50 | 42.36 | 85 | 10.7 |
| 16 | 76.04 | 51 | 41.38 | 86 | 10.03 |
| 17 | 75.04 | 52 | 40.41 | 87 | 9.38 |
| 18 | 74.05 | 53 | 39.43 | 88 | 8.76 |
| 19 | 73.05 | 54 | 38.46 | 89 | 8.16 |
| 20 | 72.06 | 55 | 37.49 | 90 | 7.6 |
| 21 | 71.06 | 56 | 36.52 | 91 | 7.06 |
| 22 | 70.07 | 57 | 35.55 | 92 | 6.55 |
| 23 | 69.07 | 58 | 34.58 | 93 | 6.07 |
| 24 | 68.08 | 59 | 33.62 | 94 | 5.6 |
| 25 | 67.08 | 60 | 32.65 | 95 | 5.13 |
| 26 | 66.09 | 61 | 31.69 | 96 | 4.65 |
| 27 | 65.09 | 62 | 30.73 | 97 | 4.18 |
| 28 | 64.1 | 63 | 29.77 | 98 | 3.7 |
| 29 | 63.11 | 64 | 28.82 | 99 | 3.24 |
| 30 | 62.11 | 65 | 27.86 | 100 | 2.79 |
| 31 | 61.12 | 66 | 26.91 | 101 | 2.36 |
| 32 | 60.13 | 67 | 25.96 | 102 | 1.94 |
| 33 | 59.13 | 68 | 25.02 | 103 | 1.59 |
| 34 | 58.14 | 69 | 24.08 | 104 | 1.28 |
|  |  |  |  | 105 | 1.02 |

SLE: standard life expectancy at age. Based on projected frontier period life expectancy and life table for year 2050, UN Population Division 2013. [3]

## Supplementary Table 3. List of WHO Member States with population size of 90,000 and over from the Region of the Americas.

| **No.** | **ISO Code** | **Country** |
| --- | --- | --- |
| 1 | ARG | Argentina |
| 2 | ATG | Antigua and Barbuda |
| 3 | BHS | Bahamas |
| 4 | BLZ | Belize |
| 5 | BOL | Bolivia, Plurinational State of |
| 6 | BRA | Brazil |
| 7 | BRB | Barbados |
| 8 | CAN | Canada |
| 9 | CHL | Chile |
| 10 | COL | Colombia |
| 11 | CRI | Costa Rica |
| 12 | CUB | Cuba |
| 13 | DOM | Dominican Republic |
| 14 | ECU | Ecuador |
| 15 | GRD | Grenada |
| 16 | GTM | Guatemala |
| 17 | GUY | Guyana |
| 18 | HND | Honduras |
| 19 | HTI | Haiti |
| 20 | JAM | Jamaica |
| 21 | LCA | Saint Lucia |
| 22 | MEX | Mexico |
| 23 | NIC | Nicaragua |
| 24 | PAN | Panama |
| 25 | PER | Peru |
| 26 | PRY | Paraguay |
| 27 | SLV | El Salvador |
| 28 | SUR | Suriname |
| 29 | TTO | Trinidad and Tobago |
| 30 | URY | Uruguay |
| 31 | USA | United States |
| 32 | VCT | Saint Vincent and the Grenadines |
| 33 | VEN | Venezuela, Bolivarian Republic of |

Two WHO Member States from the Region of the Americas —Dominica, and Saint Kiits and Nevis— are not included in the analysis because they have a population size lower than 90,000 in 2015

## Supplementary Table 4. Premature mortality from four majors noncommunicable diseases in the Region of the Americas, 2015.

### Panel A. Both sexes

| **Age group** | **deaths (a)** | **death (95% LL) (b)** | **deaths (95% UL) (c)** | **SLE (d)** | **YLL  (e) = (a) * (d)** | **YLL (95% LL)  (f) = (b) * (d)** | **YLL (95% UL)  (g) = (c) * (d)** | **Population (h)** | **YLL rate  (i) = (e)/(h) *100,000** | **YLL rate (95% LL) (j)=(f)/(h) *100,000** | **YLL rate (95% UL) (k)=(g)/(h) *100,000** | **Std pop weight  (l)** | **ASYR  (m)=(i)*(l)** | **ASYR  (95% LL)  (n)=(j)*(l)** | **ASYR  (95% UL) (o)=(k)*(l)** |
| --- | --- | --- | --- | --- | --- | --- | --- | --- | --- | --- | --- | --- | --- | --- | --- |
| 0-4 | 10,109 | 7,006 | 13,351 | 89.41 | 903,870 | 626,432 | 1,193,730 | 74,910,767 | 1,206.6 | 836.2 | 1,593.5 | 0.0886 | 106.9 | 74.1 | 141.2 |
| 5-9 | 4,844 | 3,812 | 6,160 | 84.52 | 409,448 | 322,219 | 520,654 | 75,955,736 | 539.1 | 424.2 | 685.5 | 0.0869 | 46.8 | 36.9 | 59.6 |
| 10-14 | 4,925 | 4,012 | 6,073 | 79.53 | 391,663 | 319,040 | 482,998 | 77,055,947 | 508.3 | 414.0 | 626.8 | 0.086 | 43.7 | 35.6 | 53.9 |
| 15-19 | 8,965 | 7,388 | 10,972 | 74.54 | 668,288 | 550,709 | 817,827 | 78,750,920 | 848.6 | 699.3 | 1,038.5 | 0.0847 | 71.9 | 59.2 | 88.0 |
| 20-24 | 12,334 | 10,275 | 14,987 | 69.57 | 858,048 | 714,823 | 1,042,649 | 79,725,807 | 1,076.2 | 896.6 | 1,307.8 | 0.0822 | 88.5 | 73.7 | 107.5 |
| 25-29 | 17,215 | 14,484 | 20,710 | 64.6 | 1,112,097 | 935,689 | 1,337,884 | 77,478,740 | 1,435.4 | 1,207.7 | 1,726.8 | 0.0793 | 113.8 | 95.8 | 136.9 |
| 30-34 | 26,836 | 23,009 | 31,628 | 59.63 | 1,600,214 | 1,372,043 | 1,885,955 | 73,714,765 | 2,170.8 | 1,861.3 | 2,558.4 | 0.0761 | 165.2 | 141.6 | 194.7 |
| 35-39 | 39,472 | 34,312 | 45,869 | 54.67 | 2,157,925 | 1,875,858 | 2,507,676 | 68,415,366 | 3,154.2 | 2,741.9 | 3,665.4 | 0.0715 | 225.5 | 196.0 | 262.1 |
| 40-44 | 64,154 | 56,615 | 73,399 | 49.73 | 3,190,381 | 2,815,472 | 3,650,140 | 63,794,631 | 5,001.0 | 4,413.3 | 5,721.7 | 0.0659 | 329.6 | 290.8 | 377.1 |
| 45-49 | 105,182 | 93,614 | 119,276 | 44.81 | 4,713,227 | 4,194,851 | 5,344,778 | 60,369,057 | 7,807.4 | 6,948.7 | 8,853.5 | 0.0604 | 471.6 | 419.7 | 534.8 |
| 50-54 | 181,543 | 163,157 | 203,752 | 39.92 | 7,247,186 | 6,513,220 | 8,133,772 | 57,785,174 | 12,541.6 | 11,271.4 | 14,075.9 | 0.0537 | 673.5 | 605.3 | 755.9 |
| 55-59 | 266,676 | 241,132 | 297,057 | 35.07 | 9,352,322 | 8,456,516 | 10,417,776 | 51,389,071 | 18,199.0 | 16,455.9 | 20,272.4 | 0.0455 | 828.1 | 748.7 | 922.4 |
| 60-64 | 342,106 | 310,038 | 379,983 | 30.25 | 10,348,705 | 9,378,656 | 11,494,482 | 43,427,629 | 23,829.8 | 21,596.1 | 26,468.1 | 0.0372 | 886.5 | 803.4 | 984.6 |
| 65-69 | 414,043 | 376,015 | 458,633 | 25.49 | 10,553,962 | 9,584,628 | 11,690,557 | 34,099,240 | 30,950.7 | 28,108.0 | 34,283.9 | 0.0296 | 916.1 | 832.0 | 1,014.8 |
| 70-74 | 464,968 | 422,132 | 515,202 | 20.77 | 9,657,395 | 8,767,677 | 10,700,745 | 24,645,889 | 39,184.6 | 35,574.6 | 43,418.0 | 0.0221 | 866.0 | 786.2 | 959.5 |
| 75-79 | 497,750 | 451,521 | 551,734 | 16.43 | 8,178,032 | 7,418,496 | 9,064,997 | 17,733,596 | 46,116.0 | 41,833.0 | 51,117.6 | 0.0152 | 701.0 | 635.9 | 777.0 |
| 80-84 | 527,106 | 479,244 | 582,163 | 12.51 | 6,594,092 | 5,995,339 | 7,282,855 | 12,003,732 | 54,933.7 | 49,945.6 | 60,671.6 | 0.0091 | 499.9 | 454.5 | 552.1 |
| 85+ | 944,280 | 859,498 | 1,041,327 | 7.6 | 7,176,528 | 6,532,184 | 7,914,089 | 11,519,802 | 62,297.3 | 56,704.0 | 68,699.9 | 0.0063 | 392.5 | 357.2 | 432.8 |
| **Total** | **3,932,508** | **3,557,264** | **4,372,276** |  | **85,113,383** | **76,373,852** | **95,483,564** | **982,775,869** | **8,660.5** | **7,771.2** | **9,715.7** |  | **7,426.9** | **6,646.7** | **8,354.8** |

### Panel B. Males

| **Age group** | **deaths (a)** | **death (95% LL) (b)** | **deaths (95% UL) (c)** | **SLE (d)** | **YLL  (e) = (a) * (d)** | **YLL (95% LL)  (f) = (b) * (d)** | **YLL (95% UL)  (g) = (c) * (d)** | **Population (h)** | **YLL rate  (i) = (e)/(h) *100,000** | **YLL rate (95% LL) (j)=(f)/(h) *100,000** | **YLL rate (95% UL) (k)=(g)/(h) *100,000** | **Std pop weight  (l)** | **ASYR  (m)=(i)*(l)** | **ASYR  (95% LL)  (n)=(j)*(l)** | **ASYR  (95% UL) (o)=(k)*(l)** |
| --- | --- | --- | --- | --- | --- | --- | --- | --- | --- | --- | --- | --- | --- | --- | --- |
| 0-4 | 5,510 | 3,825 | 7,256 | 89.41 | 492,610 | 341,989 | 648,741 | 38,276,457 | 1,287.0 | 893.5 | 1,694.9 | 0.0886 | 114.0 | 79.2 | 150.2 |
| 5-9 | 2,755 | 2,178 | 3,486 | 84.52 | 232,840 | 184,124 | 294,666 | 38,784,073 | 600.4 | 474.7 | 759.8 | 0.0869 | 52.2 | 41.3 | 66.0 |
| 10-14 | 2,770 | 2,267 | 3,398 | 79.53 | 220,280 | 180,286 | 270,268 | 39,346,549 | 559.8 | 458.2 | 686.9 | 0.086 | 48.1 | 39.4 | 59.1 |
| 15-19 | 5,439 | 4,524 | 6,592 | 74.54 | 405,422 | 337,197 | 491,360 | 40,100,987 | 1,011.0 | 840.9 | 1,225.3 | 0.0847 | 85.6 | 71.2 | 103.8 |
| 20-24 | 7,567 | 6,361 | 9,106 | 69.57 | 526,465 | 442,543 | 633,476 | 40,507,935 | 1,299.7 | 1,092.5 | 1,563.8 | 0.0822 | 106.8 | 89.8 | 128.5 |
| 25-29 | 10,030 | 8,498 | 11,969 | 64.6 | 647,926 | 548,997 | 773,176 | 39,126,634 | 1,656.0 | 1,403.1 | 1,976.1 | 0.0793 | 131.3 | 111.3 | 156.7 |
| 30-34 | 14,633 | 12,600 | 17,155 | 59.63 | 872,574 | 751,343 | 1,022,973 | 36,891,284 | 2,365.3 | 2,036.6 | 2,772.9 | 0.0761 | 180.0 | 155.0 | 211.0 |
| 35-39 | 20,751 | 18,079 | 24,042 | 54.67 | 1,134,482 | 988,403 | 1,314,360 | 33,950,567 | 3,341.6 | 2,911.3 | 3,871.4 | 0.0715 | 238.9 | 208.2 | 276.8 |
| 40-44 | 33,775 | 29,887 | 38,511 | 49.73 | 1,679,640 | 1,486,275 | 1,915,157 | 31,444,144 | 5,341.7 | 4,726.7 | 6,090.7 | 0.0659 | 352.0 | 311.5 | 401.4 |
| 45-49 | 56,860 | 50,774 | 64,225 | 44.81 | 2,547,915 | 2,275,205 | 2,877,924 | 29,705,332 | 8,577.3 | 7,659.2 | 9,688.2 | 0.0604 | 518.1 | 462.6 | 585.2 |
| 50-54 | 101,873 | 91,895 | 113,856 | 39.92 | 4,066,780 | 3,668,442 | 4,545,136 | 28,288,025 | 14,376.3 | 12,968.2 | 16,067.3 | 0.0537 | 772.0 | 696.4 | 862.8 |
| 55-59 | 154,633 | 140,410 | 171,476 | 35.07 | 5,422,967 | 4,924,173 | 6,013,652 | 24,883,772 | 21,793.2 | 19,788.7 | 24,167.0 | 0.0455 | 991.6 | 900.4 | 1,099.6 |
| 60-64 | 200,139 | 182,214 | 221,237 | 30.25 | 6,054,216 | 5,511,985 | 6,692,421 | 20,740,473 | 29,190.3 | 26,576.0 | 32,267.4 | 0.0372 | 1,085.9 | 988.6 | 1,200.3 |
| 65-69 | 238,451 | 217,424 | 263,027 | 25.49 | 6,078,123 | 5,542,150 | 6,704,566 | 16,035,250 | 37,904.8 | 34,562.3 | 41,811.4 | 0.0296 | 1,122.0 | 1,023.0 | 1,237.6 |
| 70-74 | 259,568 | 236,528 | 286,520 | 20.77 | 5,391,231 | 4,912,679 | 5,951,015 | 11,240,422 | 47,962.9 | 43,705.5 | 52,943.0 | 0.0221 | 1,060.0 | 965.9 | 1,170.0 |
| 75-79 | 263,623 | 239,887 | 291,288 | 16.43 | 4,331,330 | 3,941,340 | 4,785,860 | 7,853,223 | 55,153.5 | 50,187.5 | 60,941.4 | 0.0152 | 838.3 | 762.9 | 926.3 |
| 80-84 | 262,878 | 239,571 | 289,675 | 12.51 | 3,288,606 | 2,997,038 | 3,623,837 | 4,935,106 | 66,637.0 | 60,729.0 | 73,429.8 | 0.0091 | 606.4 | 552.6 | 668.2 |
| 85+ | 378,818 | 344,186 | 418,577 | 7.6 | 2,879,019 | 2,615,810 | 3,181,183 | 4,006,690 | 71,855.3 | 65,286.1 | 79,396.8 | 0.0063 | 452.7 | 411.3 | 500.2 |
| **Total** | **2,020,073** | **1,831,108** | **2,241,396** |  | **46,272,426** | **41,649,979** | **51,739,771** | **486,116,923** | **9,518.8** | **8,567.9** | **10,643.5** |  | **8,756.0** | **7,870.5** | **9,803.8** |

### Panel C. Females

| **Age group** | **deaths (a)** | **death (95% LL) (b)** | **deaths (95% UL) (c)** | **SLE (d)** | **YLL  (e) = (a) * (d)** | **YLL (95% LL)  (f) = (b) * (d)** | **YLL (95% UL)  (g) = (c) * (d)** | **Population (h)** | **YLL rate  (i) = (e)/(h) *100,000** | **YLL rate (95% LL) (j)=(f)/(h) *100,000** | **YLL rate (95% UL) (k)=(g)/(h) *100,000** | **Std pop weight  (l)** | **ASYR  (m)=(i)*(l)** | **ASYR  (95% LL)  (n)=(j)*(l)** | **ASYR  (95% UL) (o)=(k)*(l)** |
| --- | --- | --- | --- | --- | --- | --- | --- | --- | --- | --- | --- | --- | --- | --- | --- |
| 0-4 | 4,600 | 3,181 | 6,095 | 89.41 | 411,261 | 284,443 | 544,989 | 36,634,310 | 1,122.60 | 776.4 | 1,487.60 | 0.0886 | 99.5 | 68.8 | 131.8 |
| 5-9 | 2,090 | 1,634 | 2,674 | 84.52 | 176,608 | 138,095 | 225,988 | 37,171,663 | 475.1 | 371.5 | 608 | 0.0869 | 41.3 | 32.3 | 52.8 |
| 10-14 | 2,155 | 1,745 | 2,675 | 79.53 | 171,383 | 138,754 | 212,729 | 37,709,398 | 454.5 | 368 | 564.1 | 0.086 | 39.1 | 31.6 | 48.5 |
| 15-19 | 3,527 | 2,864 | 4,380 | 74.54 | 262,866 | 213,512 | 326,467 | 38,649,933 | 680.1 | 552.4 | 844.7 | 0.0847 | 57.6 | 46.8 | 71.5 |
| 20-24 | 4,766 | 3,914 | 5,881 | 69.57 | 331,583 | 272,280 | 409,174 | 39,217,872 | 845.5 | 694.3 | 1,043.30 | 0.0822 | 69.5 | 57.1 | 85.8 |
| 25-29 | 7,185 | 5,986 | 8,742 | 64.6 | 464,172 | 386,692 | 564,708 | 38,352,106 | 1,210.30 | 1,008.30 | 1,472.40 | 0.0793 | 96.0 | 80.0 | 116.8 |
| 30-34 | 12,203 | 10,409 | 14,472 | 59.63 | 727,640 | 620,701 | 862,982 | 36,823,481 | 1,976.00 | 1,685.60 | 2,343.60 | 0.0761 | 150.4 | 128.3 | 178.3 |
| 35-39 | 18,720 | 16,233 | 21,828 | 54.67 | 1,023,443 | 887,455 | 1,193,316 | 34,464,799 | 2,969.50 | 2,575.00 | 3,462.40 | 0.0715 | 212.3 | 184.1 | 247.6 |
| 40-44 | 30,379 | 26,728 | 34,888 | 49.73 | 1,510,741 | 1,329,197 | 1,734,983 | 32,350,487 | 4,669.90 | 4,108.70 | 5,363.10 | 0.0659 | 307.7 | 270.8 | 353.4 |
| 45-49 | 48,322 | 42,840 | 55,051 | 44.81 | 2,165,312 | 1,919,646 | 2,466,854 | 30,663,725 | 7,061.50 | 6,260.30 | 8,044.90 | 0.0604 | 426.5 | 378.1 | 485.9 |
| 50-54 | 79,669 | 71,262 | 89,896 | 39.92 | 3,180,406 | 2,844,777 | 3,588,637 | 29,497,149 | 10,782.10 | 9,644.20 | 12,166.00 | 0.0537 | 579.0 | 517.9 | 653.3 |
| 55-59 | 112,043 | 100,723 | 125,581 | 35.07 | 3,929,355 | 3,532,343 | 4,404,124 | 26,505,299 | 14,824.80 | 13,326.90 | 16,616.00 | 0.0455 | 674.5 | 606.4 | 756.0 |
| 60-64 | 141,967 | 127,824 | 158,746 | 30.25 | 4,294,489 | 3,866,672 | 4,802,062 | 22,687,156 | 18,929.20 | 17,043.40 | 21,166.40 | 0.0372 | 704.2 | 634.0 | 787.4 |
| 65-69 | 175,592 | 158,591 | 195,606 | 25.49 | 4,475,839 | 4,042,477 | 4,985,991 | 18,063,990 | 24,777.70 | 22,378.70 | 27,601.80 | 0.0296 | 733.4 | 662.4 | 817.0 |
| 70-74 | 205,400 | 185,604 | 228,682 | 20.77 | 4,266,165 | 3,854,998 | 4,749,730 | 13,405,467 | 31,824.10 | 28,756.90 | 35,431.30 | 0.0221 | 703.3 | 635.5 | 783.0 |
| 75-79 | 234,127 | 211,635 | 260,447 | 16.43 | 3,846,702 | 3,477,157 | 4,279,136 | 9,880,373 | 38,932.80 | 35,192.60 | 43,309.50 | 0.0152 | 591.8 | 534.9 | 658.3 |
| 80-84 | 264,227 | 239,672 | 292,487 | 12.51 | 3,305,486 | 2,998,300 | 3,659,018 | 7,068,626 | 46,762.80 | 42,417.00 | 51,764.20 | 0.0091 | 425.5 | 386.0 | 471.1 |
| 85+ | 565,462 | 515,312 | 622,751 | 7.6 | 4,297,510 | 3,916,374 | 4,732,905 | 7,513,112 | 57,200.10 | 52,127.20 | 62,995.30 | 0.0063 | 360.4 | 328.4 | 396.9 |
| **Total** | **1,912,434** | **1,726,157** | **2,130,882** |  | **38,840,961** | **34,723,873** | **43,743,793** | **496,658,946** | **7,820.4** | **6,991.5** | **8,807.6** |  | **6,272.0** | **5,583.4** | **7,095.5** |

## Supplementary Table 5. Annual Percent Change (APC) and Average Annual Percent Change (AAPC) of the age-standardized YLL rates (ASYR) by sex, Region of the Americas.

### Panel A. ASYR and 95% uncertainty intervals (95% UI) for 2000 and 2015, AAPC and % change in ASYR in the period 2000-2015, and trends statistics

| **Sex** | **Segment** | **ASYR 2000  (95% UI)** | **ASYR 2015 (95% UI)** | **APC (95% UI)** | **Test Statistic** | **P-Value** | **% Change (95% UI)** |
| --- | --- | --- | --- | --- | --- | --- | --- |
| **Both sexes** | **2000-2015** | 9,710.1  (8,852.6 - 10,693.1) | 7,427.5  (6,647.2 - 8,355.3) | -1.8  (-1.9, -1.7) | -29.7 | 0.000 | -27.0  (-28.8, -25.5) |
| **Female** | **2000-2015** | 8,182.9  (7,426.6 - 9,053.3) | 6,272.7  (5,584.0 - 7,096.2) | -1.8  (-1.9, -1.6) | -19.7 | 0.000 | -27.0  (-28.8, -24.0) |
| **Male** | **2000-2015** | 11,544.7  (10,564.1 - 12,664.8) | 8,756.6  (7,871.0 - 9,804.4) | -1.8  (-1.9, -1.7) | -28.6 | 0.000 | -27.0  (-28.8, -25.5) |

### Panel B. ASYR with 95% UI at the beginning and the end of segment, APC and % change in ASYR, and trends statistics

| **Sex** | **Segment** | **ASYR start  (95% UI)** | **ASYR end  (95% UI)** | **APC  (95% UI)** | **Test Statistic** | **P-Value** | **% Change (95% UI)** |
| --- | --- | --- | --- | --- | --- | --- | --- |
| **Both sexes** | **2000-2009** | 9,710.1  (8,852.6 - 10,693.1) | 8,116.8  (7,361.6 - 8,974.3) | -2.0  (-2.2, -1.9) | -31.9 | 0.000 | -18.0  (-19.8, -17.1) |
|  | **2009-2015** | 8,116.8  (7,361.6 - 8,974.3) | 7,427.5  (6,647.2 - 8,355.3) | -1.5  (-1.7, -1.2) | -12.4 | 0.000 | -9.0  (-10.2, -7.2) |
| **Female** | **2000-2003** | 8,182.9  (7,426.6 - 9,053.3) | 7,793.2  (7,061.9 - 8,629.2) | -1.6  (-2.2, -1.1) | -6.5 | 0.000 | -4.8  (-6.6, -3.3) |
|  | **2003-2007** | 7,793.2  (7,061.9 - 8,629.2) | 7,066.4  (6,392.1 - 7,836.1) | -2.4  (-2.9, -1.8) | -9.4 | 0.000 | -9.6  (-11.6, -7.2) |
|  | **2007-2015** | 7,066.4  (6,392.1 - 7,836.1) | 6,272.7  (5,584.0 - 7,096.2) | -1.5  (-1.6, -1.4) | -27.1 | 0.000 | -12.0  (-12.8, -11.2) |
| **Male** | **2000-2011** | 11,544.7  (10,564.1 - 12,664.8) | 9,252.5  (8,400.3 - 10,226.2) | -2.0  (-2.1, -1.9) | -45.9 | 0.000 | '-22.0  (-23.1, -20.9) |
|  | **2011-2015** | 9,252.5  (8,400.3 - 10,226.2) | 8,756.6  (7,871.0 - 9,804.4) | -1.3  (-1.7, -0.8) | -6.2 | 0.000 | '-5.2  (-6.8, -3.2) |

In both sexes combined, a joinpoint was found in 2009. In female two joinpoints were found in 2003 and 2007, while in male a joinpoint was found in 2011. APC significant different from zero at the alpha = 0.05 level. Negative sign means a decrease or reduction of ASYR per 100,000 population in corresponding segment or period of time. Test statistics refers to t-distribution test if AAPC is within one segment, otherwise it refers to normal (z) distribution test.

## References

[1] Coale A, Demeny P, Vaughan B. Regional Model Life Tables and Stable Populations, 2nd Edn. New York: Academc Press, 1983

[2] Coale A, Guo G. Revised regional model life tables at very low levels of mortality. Population Index 1989;55:613-43

[3] United Nations. World Population Prospects: The 2017 Revision, United Nations, Department of Economics and Social Affairs, Population Division. New York; 2017. Available at: <http://esa.un.org/wpp/> Accessed September 11, 2017.
